# Determination of thalidomide concentration in human plasma by liquid chromatography-tandem mass spectrometry

**DOI:** 10.3892/etm.2012.847

**Published:** 2012-11-30

**Authors:** NAN BAI, XIANG-YONG CUI, JIN WANG, CHUN-GUANG SUN, HE-KUN MEI, BEI-BEI LIANG, YUN CAI, XIU-JIE SONG, JING-KAI GU, RUI WANG

**Affiliations:** 1 The Center of Medicine Clinical Research, Chinese PLA General Hospital, Beijing 100853;; 2 Research Center for Drug Metabolism, Jilin University, Changchun 130023, P.R. China

**Keywords:** human plasma, thalidomide, multiple reaction monitoring, liquid chromatography-tandem mass spectrometry

## Abstract

A rapid, sensitive and specific analytical method based on high-performance liquid chromatography-tandem mass spectrometry (LC-MS/MS) has been developed for the determination of thalidomide concentration in human plasma. The analyte and internal standard were extracted by liquid-liquid extraction with ether-dichloromethane (3:2, v/v) and separated on a TC-C_18_ column using methanol-10 mM ammonium acetate-formic acid (60:40:0.04, v/v/v) as the mobile phase at a flow rate of 0.9 ml/min. The detection was performed using an API 4000 triple quadrupole mass spectrometer in the positive electrospray ionization (ESI) mode and completed within 3.0 min. The multiple reaction monitoring (MRM) transitions were m/z 259.1→84.0 for the analyte and m/z 195.9→138.9 for temozolomide. The calibration curve exhibited a linear dynamic range of 2–1500 ng/ml (r>0.9991). The intra-and inter-day precisions (as relative standard deviation; RSD) were 6.8–13.5% and 4.3–5.0% respectively and the accuracy (as relative error; RE) was 2.0–3.5%. The recoveries and matrix effects were satisfactory in all the biological matrices examined. This method was successfully used in a pharmacokinetic study of thalidomide in healthy male volunteers receiving an oral administration of a 200-mg dose.

## Introduction

Thalidomide [(±) α-(N-phthalimido)-glutarimide] is derived from glutamic acid and was initially synthesized in 1954 in West Germany. It was prescribed as a safer, non-barbiturate sedative-hypnotic and used for treating morning sickness in pregnant women for a number of years in Europe until it was withdrawn in 1961 for its teratogenicity. Thalidomide has since been revealed to have a wide range of pharmacological effects, particularly anti-inflammatory and immunomodulatory activities (1,2). Therefore, it has been evaluated for the management of numerous inflammatory and autoimmune diseases, including multiple myeloma (3–5), rheumatoid arthritis (6,7), ankylosing spondylitis (8–10), Crohn’s disease (11,12), lupus erythematosus (13) and Behçet’s disease (14). Based on its confirmed efficacy, thalidomide was approved by the Food and Drug Administration (FDA) for the treatment of erythema nodosum leprosum in 1998 and multiple myeloma in 2006. In China, the State Food and Drug Administration (SFDA) approved thalidomide for the treatmeat of ankylosing spondylitis in 2008, therefore, analysis of the pharmacokinetic parameters of thalidomide is necessary.

Several methods have been reported for the quantitation of thalidomide in biological fluids, including high-performance liquid chromatography (HPLC) (15–23) and liquid chromatography tandem mass spectrometry (LC-MS/MS) (24,25). The present study reports a quantitative method for the determination of thalidomide concentration in biological fluids using LC-MS/MS with temozolomide as the internal standard (IS). The method was fully validated and successfully applied to a pharmacokinetic study in healthy volunteers.

## Materials and methods

### Chemicals and reagents

**Figure 1.**
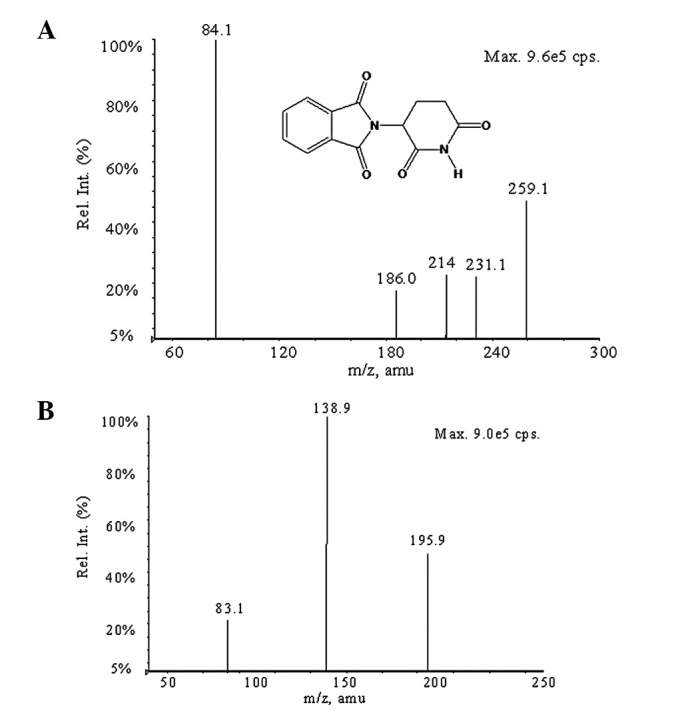
Full-scan product ion spectra of [M+H]^+^ for (A) thalidomide and (B) temozolomide.

Thalidomide (purity >99%) was provided by Changzhou Pharmaceutical Co. Ltd. (Changzhou, China). Temozolomide acid (purity >99%) was provided by the National Institute for the Control of Pharmaceutical and Biological Products (Beijing, China). The structure of thalidomide is shown in Fig. 1. Methanol and acetonitrile (HPLC grade) were purchased from Fisher Scientific (Fair Lawn, NJ, USA). Distilled water was prepared from deionized water. All other chemicals and solvents (analytical grade) were used without further purification. Blank (drug free) human plasma was obtained from the Changchun Blood Donor Service (Changchun, China).

### Instrumentation

The Agilent 1100 series (Agilent, Palo Alto, CA, USA) HPLC system consisted of a pump, an autosampler and a column oven. The mass spectrometric detection employed an Applied Biosystems Sciex API 4000 mass spectrometer (Applied Biosystems Sciex, Mississauga, ON, Canada) equipped with an electrospray ionization (ESI) source. Analytical software (Applied Biosystems/MDS Sciex, version 1.3) was used for the data acquisition and processing.

### LC-MS/MS conditions

A TC-C_18_ analytical column (50x4.6 mm, 5 *μ*m; Agela, Wilmington, DE, USA) was used in the study. The isocratic mobile phase was methanol-10 mM ammonium acetate-formic acid (60:40:0.04, v/v/v) with a flow rate of 0.9 ml/min and the post-column splitting ratio was 1:1. A 20-*μ*l aliquot of the sample was injected into the LC-MS/MS system for analysis. The column temperature was maintained at 40°C.

All measurements were performed with the mass spectrometer operated in the positive ESI mode. The multiple reaction monitoring (MRM) transitions were m/z 259.1→84.0 for thalidomide and m/z 195.9→138.9 for temozolomide.

Other parameters were as follows: collision gas, curtain gas, gas 1 and gas 2 (nitrogen) pressures, 15, 15, 55 and 55 psi respectively; dwell time, 200 msec; ion spray voltage, 5000 V; source temperature, 500°C; declustering potential (DP), 37 V for thalidomide and 31 V for temozolomide; and collision energy (CE), 20 eV for thalidomide and 11 eV for temozolomide. Unit resolution was used for Q1 and Q3 mass detection.

### Preparation of calibration standard samples and quality control samples

The standard stock solution (1 mg/ml) of thalidomide was prepared by dissolving thalidomide (25 mg) in 25 ml methanol-acetonitrile-formic acid (50:49:1, v/v/v). The calibration standard samples were prepared at concentrations of 2, 5, 15, 50, 150, 500 and 1500 ng/ml with the same mixed solvent. Quality control (QC) solutions with low, medium and high concentrations (5, 50 and 1200 ng/ ml) were prepared in the same manner. The standard IS stock solution (1 mg/ml) of temozolomide was prepared by dissolving temozolomide (25 mg) in 25 ml methanol and the IS working solution (100 ng/ml) was prepared with methanol-10 mM ammonium acetate (50:50, v/v; the latter included 2% formic acid).

The frozen plasma samples were thawed at room temperature and vortex-mixed with an equal volume of 0.025 M Sorensen’s citrate buffer (pH 1.5) to prevent spontaneous hydrolysis (21). All solutions were stored at 4°C prior to use.

### Sample preparation

A 100-*μ*l aliquot of the plasma was transferred to a micro-centrifuge tube to which 100 *μ*l IS working solution and 150 *μ*l methanol-ammonium acetate (50:50, v/v; the latter included 0.2% formic acid) were also added and then vortex-mixed. The mixture was extracted with 3 ml ether-dichloromethane (3:2, v/v) by agitation for 10 min. After centrifuging at 3000 x g for 5 min, the organic phase was separated and evaporated until dry at 40°C under a gentle stream of nitrogen. The residue was reconstituted in 150 *μ*l of the mobile phase, of which 20 *μ*l was injected into the LC-MS/MS system. The samples with concentrations greater than the maximum standard in the calibration curve were determined by dilution of these samples with blank plasma.

### Assay validation

According to the FDA guidelines for the validation of bioanalytical methods (26), the method was fully validated with regard to the specificity, linearity and sensitivity, matrix effects and extraction recovery, accuracy and precision, the stability and the effect of dilution.

To determine the specificity of the assay, six replicates of the pooled blank human plasma were analyzed to investigate potential interference around the chromatography peak region for the analyte and IS.

Linearity was assessed by three independent calibration curves, each based on seven spiked plasma samples with concentrations in the range of 2–1500 ng/ml. The calibration curves were analyzed by the ratio of the peak area of thalidomide and IS with 1/χ^2^ weighted least squares linear regression analysis (χ = concentration).

Intra- and inter-day precision [relative standard deviation (RSD)] were determined by assaying six replicates of the QC samples at 5, 50 and 1200 ng/ml on three different days. Accuracy [relative error (RE)] was determined on the basis of the total data set (n=18). Intra- and inter-day precisions calculated as RSD (%) were required to be <15% and accuracy as RE (%) was required to be within ±15%. The lower limit of quantitation (LLOQ) was defined as the lowest concentration that could be determined with acceptable precision (±20%) and accuracy (±20%).

The recoveries of the analyte and IS were determined by comparing the peak areas of extracted standard samples with the peak areas of post-extraction plasma blanks spiked at the corresponding concentrations. The matrix effects and recovery of the analyte were assessed similarly using four matrix replicates spiked with QC samples and IS (100 ng/ml). The matrix effects of the analyte and IS were evaluated by comparing the peak areas of post-extraction blank plasma spiked at the QC sample concentrations with the areas obtained by direct injection of the corresponding standard solutions.

The long-term, freeze-thaw and post-processing stability were evaluated using QC samples after storage at −20°C for 1 month, after three freeze/thaw cycles and after storage in reconstitution solutions in the autosampler at room temperature for 4 h, respectively.

The effect of dilution was evaluated for the analysis of plasma samples containing analyte at concentrations higher than the upper limit of the standard curve by analyzing three replicates of plasma spiked with analyte at two-fold dilutions of the three QC concentrations (10, 100 and 2400 ng/ml) and diluting with blank plasma to three concentration levels (5, 50 and 1200 ng/ml).

### Pharmacokinetic study

The method was applied to a single 200-mg dose study of thalidomide in 10 healthy male volunteers who gave informed consent prior to the clinical trial The study was approved by the Ethics Committee of the Chinese PLA General Hospital (Beijing, China). After a 10-h fast, the volunteers received a single tablet containing 200 mg thalidomide. Blood samples (4 ml) were collected prior to dosage and at 0.5, 1.0, 1.5, 2.0, 3.0, 4.0, 6.0, 8.0, 12.0, 16.0, 24.0, 36.0, 48.0 and 72.0 h after dosing. After centrifuging at 3000 x g for 10 min, the plasma samples were transferred to tubes with an equal volume of 0.025 M Sorensen’s citrate buffer pH 1.5 and stored at −80°C. The pharmacokinetic parameters were calculated using WinNolin 5.2.1.

## Results and Discussion

### Mass spectrometry and chromatography

The analyte and IS response was superior under the positive ionization mode. In this mode, the soft ionization process in the Turbo Ion Spray (TIS) source produces the precursor ions [M+H]^+^. The MS-MS detector was operated at unit resolution in the MRM modes using the transitions of the protonated molecular ions of analyte at m/z 259.1→84.0 and IS at m/z 195.9→138.9. The full product ion spectra of the analyte and IS are shown in Fig. 1.

Several commercial HPLC columns were evaluated in the present study, including the Nucleosil C_18_ (5 *μ*m, 50x4.6 mm), Hypersil ODS2 (5 *μ*m, 150x4.6 mm), Restek Pinnacle C_18_ (3 *μ*m, 100x2.1 mm), TC-C_18_ (5 *μ*m, 50x4.6 mm) and Zorbax Extend C_18_ (5 *μ*m, 150x4.6 mm). Of these columns, the TC-C_18_ column was noted to yield the best chromatograms with minimal matrix effects. Under the optimum assay conditions, the analyte and IS were free of interference from endogenous substances and the retention times of thalidomide and the IS (2.82 and 2.28 min, respectively) were short enough to enable quick analysis.

Two mobile phase systems with acetonitrile and methanol as the organic phase were compared in the present study. The results showed that higher sensitivity and improved peak shapes were acquired with the methanol system. Formic acid and 10 mM ammonium acetate were employed in the mobile phase, which improved the response and the peak shape. The flow rate of 0.9 ml/min gave an acceptable pressure and the split ratio of 1:1 improved the peak shape.

In a previous study, a derivative of thalidomide was used as the IS to determine the concentration of thalidomide in plasma (24). However, the derivative was not commercially available, which precluded its use in the present study. Temozolomide was used as the IS in the current study, and had a similar retention behavior and extraction recovery to thalidomide under the aforementioned conditions.

### Assay validation

**Figure 2.**
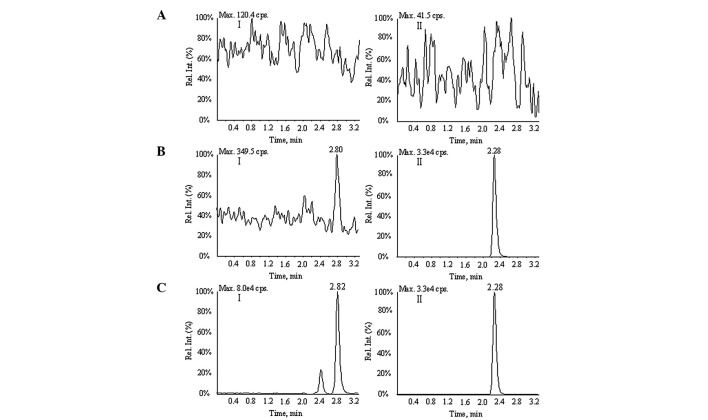
Representative MRM chromatograms of thalidomide in plasma. (A) Blank plasma; (B) blank plasma spiked with thalidomide (2 ng/ml) or internal standard (100 ng/ml); (C) plasma sample 1 h after the oral administration of a 200-mg dose. I, thalidomide; II, temozolomide; MRM, multiple reaction monitoring.

Fig. 2 shows the representative LC-MS/MS chromatograms obtained from the analysis of blank human plasma, human plasma spiked with thalidomide at 2 ng/ml and a human plasma sample obtained 1 h after the oral administration of a thalidomide tablet (200 mg). The analysis of the blank plasma samples from six different sources did not show any interference at the retention times of thalidomide (2.80 min) and IS (2.28 min) and demonstrated the specificity of this method.

The calibration curves were linear over the concentration range of 2–1500 ng/ml with a correlation coefficient of r>0.9991.

Table I shows a summary of the intra- and inter-day precision and accuracy data for the LLOQ and QC samples containing thalidomide. The intra- and inter-day precisions ranged between 4.30 and 13.51% at three QC concentrations (5, 50 and 1200 ng/ml). The intra- and inter-day RE values for thalidomide were 1.98 to 3.54% at three QC levels (5, 50 and 1200 ng/ml). These results indicated that the present method had an acceptable precision and accuracy. The LLOQ was set at 2 ng/ml for thalidomide using 100 *μ*l human plasma. The intra-day RSD, inter-day RSD and RE at the LLOQ level were 5.23, 7.78 and 3.27%, respectively.

The extraction recoveries of thalidomide at concentrations of 5, 50 and 1200 ng/ml were 92.1±4.2, 93.3±2.3 and 95.3±1.5%, respectively. The matrix effects were minimal and the results were 91.6±4.1, 92.0±2.3 and 93.4±1.4% based on nominal concentrations of 5, 50 and 1200 ng/ml, respectively.

The results of the stability evaluation in human plasma are summarized in Table II. The stability experiment indicated that thalidomide underwent no significant degradation during processing (three freeze-thaw cycles), sample storage (at room temperature for 4 h and at −20°C for 1 month) and post-treatment (in the reconstituted extract at room temperature for 24 h).

The precision (RSD) and accuracy (RE) for the measured thalidomide concentrations at 10, 100 and 2400 ng/ml following a 2-fold dilution with blank human plasma were 4.16, 2.86 and 0.99%, and 5.39, 1.78 and 1.5%, respectively.

### Pharmacokinetic study

**Figure 3.**
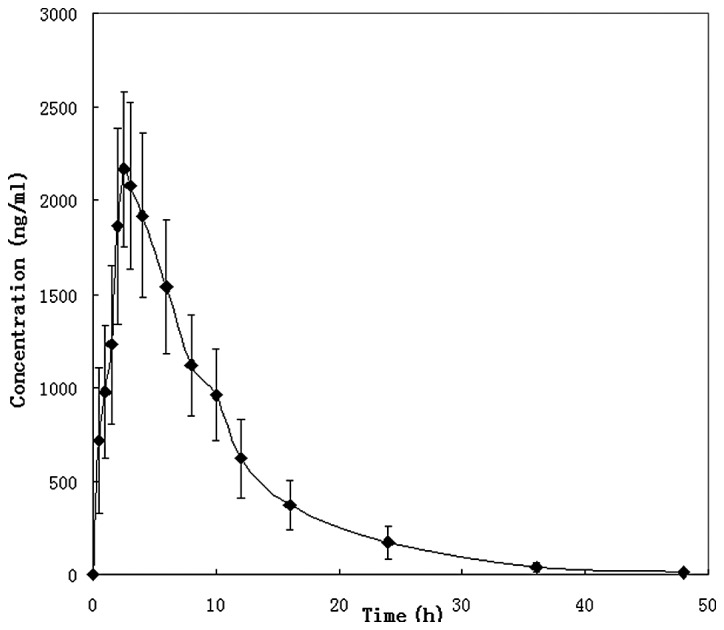
Mean plasma concentration-time profile for 200 mg thalidomide.

This method was successfully applied to the pharmacokinetic study of thalidomide in healthy male volunteers. The present study was the first to evaluate the pharmacokinetic properties of thalidomide in Chinese individuals. The mean plasma concentration-time profile of thalidomide is shown in Fig. 3. The pharmacokinetic parameters for thalidomide are as follows: C_max_, 2.40±0.26 *μ*g/ml; t_max_, 2.40±0.32 h; AUC_0−∞_, 21.62±4.01 *μ*g/h/ml; and t_1/2_, 6.18±0.84 h. The results show that the pharmacokinetic parameters for thalidomide in the present study are consistent with those from previous studies in other countries corrected for the same dose. In a previous study of 17 non-obese male volunteers, the C_max_ was 2.00±0.55 g/ml, t_max_ was 3.2±1.4 h, AUC_0−∞_ was 19.8±3.61 *μ*g/h/ml and t_1/2_ was 6.17±2.56 h (18).

A rapid and sensitive LC-MS/MS assay for the determination of thalidomide concentration in human plasma was developed and validated in the present study, which demonstrated good precision, simplicity, sensitivity and a wide range of linear concentrations with a short analysis time. The method was successfully applied to the pharmacokinetic study of thalidomide. This is the first study to report the pharmacokinetic properties of thalidomide in Chinese individuals.

## Figures and Tables

**Table I.** LLOQ (n=4) and precision and accuracy results for thalidomide in human plasma (n=18).

Nominal concentration (ng/ml)	Calculated concentration, mean ± SD (ng/ml)	Inter-day RSD (%)	Intra-day RSD (%)	RE (%)
2	2.065±0.161	5.23	7.78	3.27
5	5.235±0.19	4.68	7.54	1.98
50	53.28±2.05	4.3	13.51	3.48
1200	1274±26.55	4.99	6.84	3.54

LLOQ, lower limit of quantitation; RSD, relative standard deviation (precision); RE, relative error (accuracy).

**Table II.** Stability studies for thalidomide (4 samples each concentration).

Stability experiment	Storage condition	Nominal concentration (ng/ml)	Calculated concentration, mean ± SD (ng/ml)	RE (%)	RSD (%)
Freeze/thaw stability	After third freeze/thaw cycle at −80°C	5	4.593±0.10	0.93	1.98
		50	52.80±2.91	5.59	5.51
		1200	1189±10.02	1.00	0.84
Long-term stability	For 6 months at −80°C	5	5.166±0.08	3.33	1.58
		50	50.39±0.82	0.79	1.63
		1200	1201±21.01	0.05	1.75
Room temperature stability	Room temperature for 4 h	5	5.297±0.19	5.94	3.56
		50	56.75±0.38	13.50	0.67
		1200	1269±75.08	5.78	5.92

RE, relative error (accuracy); RSD, relative standard deviation (precision).
